# The first complete mitochondrial genome assembly and comparative analysis of the fern Blechnaceae family: *Blechnopsis orientalis*

**DOI:** 10.3389/fpls.2025.1534171

**Published:** 2025-03-20

**Authors:** Yutong Huang, Yanping Xing, Wenxiao Men, Hefei Xue, Wenjuan Hou, Yanchang Huang, Deqiang Dou, Tingguo Kang, Yanyun Yang, Liang Xu

**Affiliations:** School of Pharmacy, Liaoning University of Traditional Chinese Medicine, Dalian, China

**Keywords:** *Blechnopsis orientalis*, fern, lycophyte, mitochondrial genome, phylogeny

## Abstract

**Introduction:**

*Blechnopsis orientalis* (L.) C. Presl is a medicinal and edible fern species belonging to the Blechnaceae family. Currently, the complete mitochondrial genome of *B. orientalis*, as well as those of other Blechnaceae species, remains unreported, and studies on fern mitochondrial genome are limited.

**Methods:**

In this study, the *B. orientalis* mitochondrial genome was sequenced using both Nanopore PromethION and Illumina NovaSeq 6000 platforms. Genome annotation was performed using MITOFY and MFANNOT, with structural visualization via OGDRAW. In-depth analyses were conducted, including assessments of non-synonymous/synonymous mutation ratios (Ka/Ks), codon usage bias, repeat sequence identification, RNA editing site prediction, collinearity, and the identification of homologous fragments between chloroplast and mitochondrial genomes. Finally, we employed both the maximum likelihood (ML) and Bayesian (BI) methods to analyze the phylogenetic relationships among *B. orientalis* and nine other fern and lycophyte species.

**Results:**

The mitochondrial genome of *B. orientalis* has a complex structure comprising 80 contigs, with a total length of 501,663 bp and a GC content of 48.53%. A total of 179 genes were identified, including 40 protein-coding genes (PCGs), 98 tRNA genes, 40 rRNA genes, and one pseudogene (*rps11*). Phylogenetic analysis based on PCGs from both chloroplast genome and mitochondrial genome aligned with the relationships described in the Pteridophyte Phylogeny Group I (PPG I) system. Further comparison with mitochondrial genome of ten other reported fern and lycophyte species revealed that the mitochondrial genome PCGs in these plants are highly conserved, despite significant genome rearrangements among mitochondrial genome.

**Discussion:**

The findings of this study provide valuable insights into the evolutionary analysis of *B. orientalis* and contribute to understanding the characteristics and evolutionary relationships of mitochondrial genome in ferns and lycophytes.

## 1 Introduction

*Blechnopsis orientalis* (L.) C. Presl is a fern species belonging to the genus *Blechnopsis* within the Blechnaceae family. The genus *Blechnopsis* encompasses over 200 species, which are pantropically distributed, predominantly occurring in southern China and Southeast Asia. These ferns typically thrive in warm and humid hillside shrublands or beneath sparse forest canopies. In China, *B. orientalis* is the sole representative species of this genus (Wang et al., 2013). Notably, *B. orientalis* demonstrates remarkable resilience, capable of flourishing even in environments characterized by severe pollution (Zhu et al., 2013).

In certain southern provinces of China, the dried rhizomes and petiole remnants of *B. orientalis* are utilized as a traditional Chinese medicine known as Guan Zhong. This herbal remedy is employed to treat various ailments including wind-cold, hemoptysis, and tapeworm and roundworm infections (Lai et al., 2010). In several Southeast Asian countries, it is commonly applied to address wounds, blisters, abscesses, ulcers, as well as stomach pain and bladder discomfort (Ahmad and Holdsworth, 2003). Contemporary pharmacological studies have revealed that *B. orientalis* contains diverse bioactive compounds, such as polyphenols and flavonoids, which demonstrate antioxidant, antibacterial, and anti-inflammatory properties (Dvorakova et al., 2024). This renders it a natural source of antibacterial and antioxidant agents (Lai et al., 2017). Additionally, the young leaves of *B. orientalis* are edible as wild vegetables, making it one of the most popular ferns in Asia (Liu et al., 2012a; Giri and Uniyal, 2022).

Mitochondria serve a dual function in angiosperms, providing essential cellular energy and playing a pivotal role in plant growth and development (Van Aken and Van Breusegem, 2015). Furthermore, as an extrachromosomal genetic system, mitochondria contain highly conserved functional gene sequences (Palmer and Herbon, 1988). These sequences exhibit unique conservation patterns and evolutionary rates that differ from those of nuclear genes. Consequently, mitochondria serve as an invaluable resource for investigations in molecular evolution and molecular ecology (Janouškovec et al., 2013). An increasing number of plant mitochondrial genomes have been reported, which have actively promoted research on mitochondrial genome structure, population genetics, and evolutionary applications. For example, the mitochondrial genome of *Angelica biserrata* revealed a unique multi-branch conformation in its mtDNA structure (Wang L, et al., 2024). Mitochondrial genome studies have also helped resolve the phylogenetic position of the *Selaginella sinensis* group, shedding light on the phylogeny of the challenging Lycopodiaceae family (Tang et al., 2023). The report on the mitochondrial genome of *Angelica dahurica* provides a valuable reference for future molecular breeding efforts in *A. dahurica* and other plant species (Li et al., 2024).

Recent advancements in sequencing technologies have facilitated the analysis of an increasing number of plant genomes. However, studies on complete mitochondrial genomes of plants remain relatively limited, particularly for ferns and lycophytes. The National Center for Biotechnology Information (NCBI) database (https://www.ncbi.nlm.nih.gov/) currently contains fewer than one thousand complete mitochondrial genome sequences for plants (Wang J, et al., 2024), with only a small number available for ferns and lycophytes. These include species such as *Dryopteris crassirhizoma*, *Ophioglossum californicum*, and *Psilotum nudum* (Song et al., 2021; Guo et al., 2017). These mitochondrial genome data serve multiple purposes, including the construction of molecular markers, assessment of genetic diversity, and investigation of phylogenetic and evolutionary relationships (Zhang et al., 2024). Consequently, the assembly and annotation of *B. orientalis* mitochondrial genome can provide valuable genomic information for genetic identification and phylogenetic studies of *B. orientalis* and other ferns and lycophytes.

## 2 Materials and methods

### 2.1 Plant materials and DNA sequencing

*B. orientalis* specimens were collected in Guangzhou, Guangdong Province, China (113°21’29.40” E, 23°9’20.11” N). Fresh, healthy young leaves were selected for this study. High-quality genomic DNA was extracted from the leaves utilizing a modified Cetyl Trimethyl Ammonium Bromide (CTAB) method. Following library preparation with SQK-LSK109, DNA sequencing was performed using the Nanopore PromethION sequencing platform (Nanodrop Technologies, Wilmington, DE, USA), generating raw sequence data. The data were subsequently filtered and processed using NanoFilt and NanoPlot from the Nanopack toolkit (De Coster et al., 2018). Concurrently, a high-quality DNA library with an average fragment length of 350 bp was prepared using the Nextera XT DNA Library Preparation Kit. Sequencing was conducted on the Illumina NovaSeq 6000 platform (Illumina, San Diego, CA, USA), generating raw sequence data, which were then processed using NGS QC Toolkit (v2.3.3) (Patel and Jain, 2012).

### 2.2 Assembly and annotation of mitochondrial genome

Minimap2 (v2.15-r905) was initially employed to align the third-generation sequencing data with reference sequences (*D. crassirhizoma*: MW732172; *Lycopodium japonicum*: NC080981; *Psilotum nudum*: KX171638-KX171639; *Haplopteris ensiformis*: OM867545-M867553). The aligned sequences were then assembled using Miniasm (v0.3-r179) to obtain preliminary results. These initial assemblies were subsequently refined with NextPolish (v1.3.1). Bowtie2 (v2.3.5.1) was utilized to align second-generation sequences to the corrected assembly, followed by Unicycler (v0.4.8) to assemble the aligned second-generation data into contigs, which were visualized using Bandage (Wick et al., 2017). Minimap2 was then reapplied to align third-generation data with the contig sequences, and Bandage was used to assess contig connectivity. Manual curation was performed to finalize the results, culminating in the complete assembly of *B. orientalis* mitochondrial genome.

The mitochondrial genome of *B. orientalis* was annotated utilizing MITOFY (Alverson et al., 2010) and MFANNOT (Gautheret and Lambert, 2001), referencing previously identified angiosperm mitochondrial genome sequences from the NCBI database. The circular structure of *B. orientalis* mitochondrial genome was visualized using the OGDRAW program (Greiner et al., 2019). The complete assembled sequence of *B. orientalis* mitochondrial genome has been deposited in GenBank (NCBI) and is accessible under the accession number: PQ143019.

### 2.3 Non-synonymous/synonymous mutation ratio (Ka/Ks) analysis

The mitochondrial genome sequences for the additional species utilized in this analysis were obtained from the NCBI database. These species encompassed five ferns: *Haplopteris ensiformis* (OM867545-OM867553), *Ophioglossum californicum* (NC030900), *Ophioglossum vulgatum* (NC065260), *Psilotum nudum* (KX171638-KX171639), and *Dryopteris crassirhizoma* (MW732172); as well as five lycophytes: *Selaginella moellendorffii* (JF338143-JF338147), *Phlegmariurus squarrosus* (NC017755), *Huperzia crispata* (NC071971), *Lycopodium japonicum* (NC080981), and *Phylloglossum drummondii* (NC086557).

Homologous gene pairs were identified through pairwise grouping of the selected species. These gene pairs were then aligned using MAFFT (v7.427, https://mafft.cbrc.jp/alignment/software/). Subsequently, KaKs_Calculator (v2.0, https://sourceforge.net/projects/kakscalculator2/) was employed to calculate the non-synonymous (Ka) and synonymous (Ks) substitution rates for each gene pair using the MLWL method. The resulting Ka/Ks ratios for each gene pair were compiled, and box plots were generated using the ggplot2 package in R to visualize the data.

### 2.4 Codon preference analysis

The protein-coding sequences of the genome were extracted using PhyloSuite software. The codon usage of *B. orientalis* mitochondrial genome was analyzed using CodonW (v1.4.4, http://codonw.sourceforge.net), including the calculation of relative synonymous codon usage (RSCU) values. CUSP and CodonW were employed to determine the coding sequence (CDS) using bias parameters (Sharp and Li, 1986; Rice et al., 2000), such as the effective number of codon (ENC), GC content at GC1, GC2, and GC3 codon positions, GCall, and GC content at GC3s. To visualize codon usage, a scatter plot (ENC-plot) was generated with GC3 as the x-axis and ENC as the y-axis. The theoretical ENC value was computed using the formula: ENC= 2+GC3 + 29/[GC3^2^+(1-GC3)^2^]. Subsequently, a standard curve was plotted with GC3 as the x-axis and the theoretical ENC as the y-axis. To further compare theoretical ENC values with observed values, an ENC ratio was calculated using the formula: ENC_ratio_=(ENC_theory_ -ENC_practice_)/ENC_theory_. The frequency distribution of the ENC ratio was then obtained. Additionally, a neutral plot was created with GC3 as the x-axis and GC12 (average of GC1 and GC2) as the y-axis, incorporating a reference line representing the y=x function for subsequent analysis.

### 2.5 Repeat sequence analysis

Simple sequence repeats (SSRs) were identified utilizing MISA software (v1.0, parameters: 1-10, 2-5, 3-4, 4-3, 5-3, 6-3) (Beier et al., 2017). Tandem repeats were detected using TRF software (trf409.linux64, parameters: 2 7 7 80 10 50 2000 -f -d -m) (Benson, 1999). Dispersed repeats were analyzed using BLASTn software (v2.10.1, parameters: -word_size 7, e-value 1e-5, with redundancy and tandem repeats removed) (Chen et al., 2015). The resulting data were visualized using the ggplot2 package in R.

### 2.6 RNA editing sites

RNA editing sites in PCGs from mitochondrial genome were identified using PmtREP (Lenz et al., 2018). The prediction of RNA editing site employed a cutoff value of 0.2.

### 2.7 Collinearity analysis

Homologous sequences within mitochondrial DNA were identified using BLASTn (parameters: -evalue 1e-5 and -word_size 7) (Altschul et al., 1990). Subsequently, a custom script was utilized for visualization and calculation of homology statistics.

### 2.8 Identification of homologous fragment

Homologous sequences between the chloroplast genome and mitochondrial genome were identified using BLASTn with an E-value threshold of 1e-5, while other parameters were maintained at default settings. The results were visualized using Circos (v0.69-5) (Zhang et al., 2013). Chloroplast genome data were obtained from previous research conducted by our group, accessible under the NCBI accession number: PP533479.

### 2.9 Phylogenetic tree analysis

The species sequence information of ferns and lycophytes that possess both chloroplast and mitochondrial genomes was screened and retrieved from the NCBI database. The accession numbers for the chloroplast genomes are as follows: *B. orientalis*: PP533479, *D. crassirhizoma*: NC050008, *P. nudum*: NC003386, *O. californicum*: NC020147, *O. vulgatum*: MZ066610, *H. ensiformis*: NC065981, *L. japonicum*: NC085262, *H. squarrosa*: ON773236, *H. crispata*: NC064991, *S. moellendorffii*: HM173080, and *Andreaea regularis*: NC070057. The mitochondrial genome accession number for *A. regularis* is NC068856, while the mitochondrial genome accession numbers for the other species correspond to those utilized in the Ka/Ks analysis.

The chloroplast genome and mitochondrial genome analyses were conducted using identical methodologies, employing CDS to construct a maximum likelihood phylogenetic tree. Homologous gene sequences from various species were aligned using MAFFT (v7.427, in –auto mode) (Katoh and Standley, 2013). The aligned sequences were subsequently concatenated and trimmed using trimAl (v1.4.rev15, parameter: -gt 0.7) (Capella-Gutiérrez et al., 2009). Following trimming, the optimal evolutionary model was determined using jModelTest (v2.1.10) (Posada, 2008), which identified the GTR model. Finally, a maximum likelihood (ML) phylogenetic tree was constructed using RAxML (v8.2.10, https://cme.h-its.org/exelixis/software.html) with the GTR model and 1000 bootstrap replicates. The Bayesian (BI) phylogenetic trees were constructed separately using the common CDS of chloroplast genome and mitochondrial genome. The dataset and the sequence alignment and trimming methods were consistent with those used for the construction of the maximum likelihood (ML) phylogenetic tree. MrBayes (v3.2.7) (Huelsenbeck and Ronquist, 2001) was used to perform the Markov Chain Monte Carlo (MCMC) method with 1 million iterations, sampling every 100 generations. The first 25% of the trees were discarded as burn-in, and the final majority-rule consensus tree was generated.

## 3 Results

### 3.1 Structure and characteristics of mitochondrial genome

**Figure 1:**
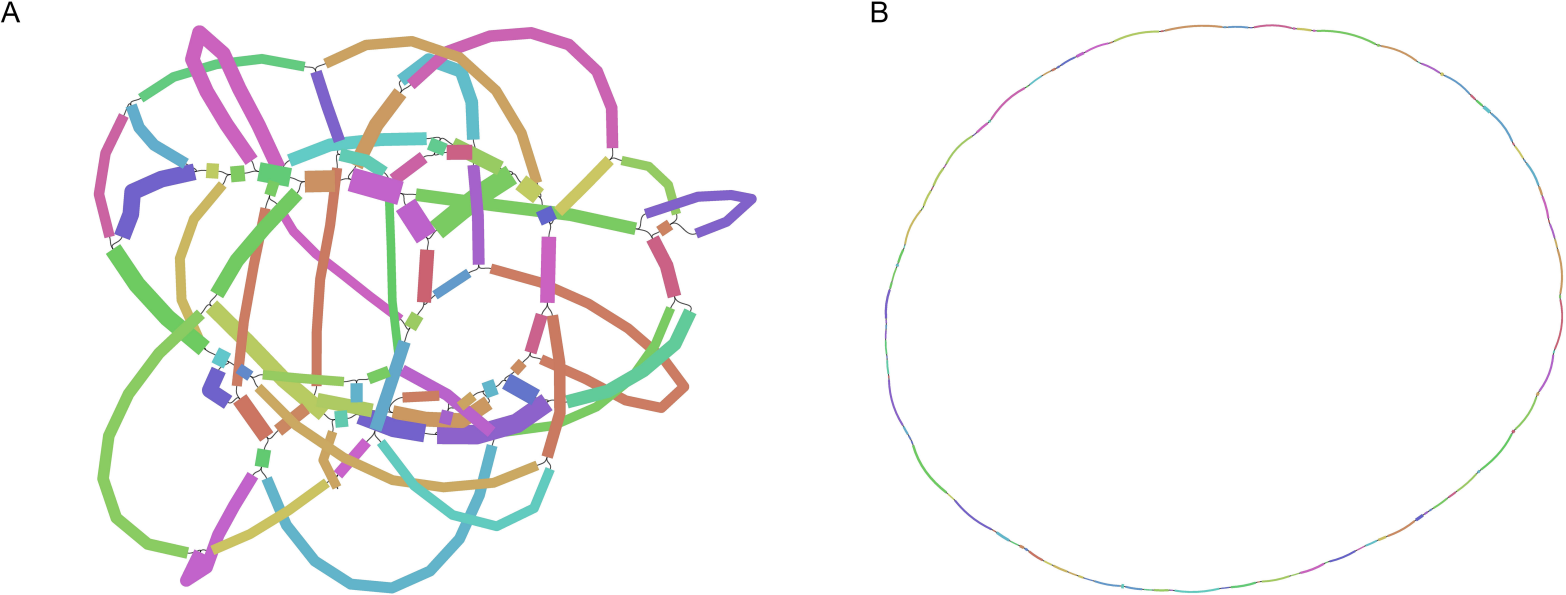
The assembly results of *B*. *orientalis* mitochondrial genome. **(A)** The image represents a GFA graph supported by long-read Nanopore data, constructed from a closed structure consisting of 80 contigs. It was visualized using the Bandage software. **(B)** Based on the connectivity and depth relationships, the closed structure formed by the 80 contig connections can be unraveled into a complete circular genome.

The mitochondrial genome of *B. orientalis* exhibits a complex, network-like dynamic structure (**Figure 1A** shows the GFA map supported by third-generation Nanopore data, which presents the network’s dynamic structure). Based on the connections and depth relationships supported by the third-generation data, we reconstructed the 80 contigs into a closed structure, which was further inferred to represent a complete circular genome (**Figure 1B**).

**Figure 2:**
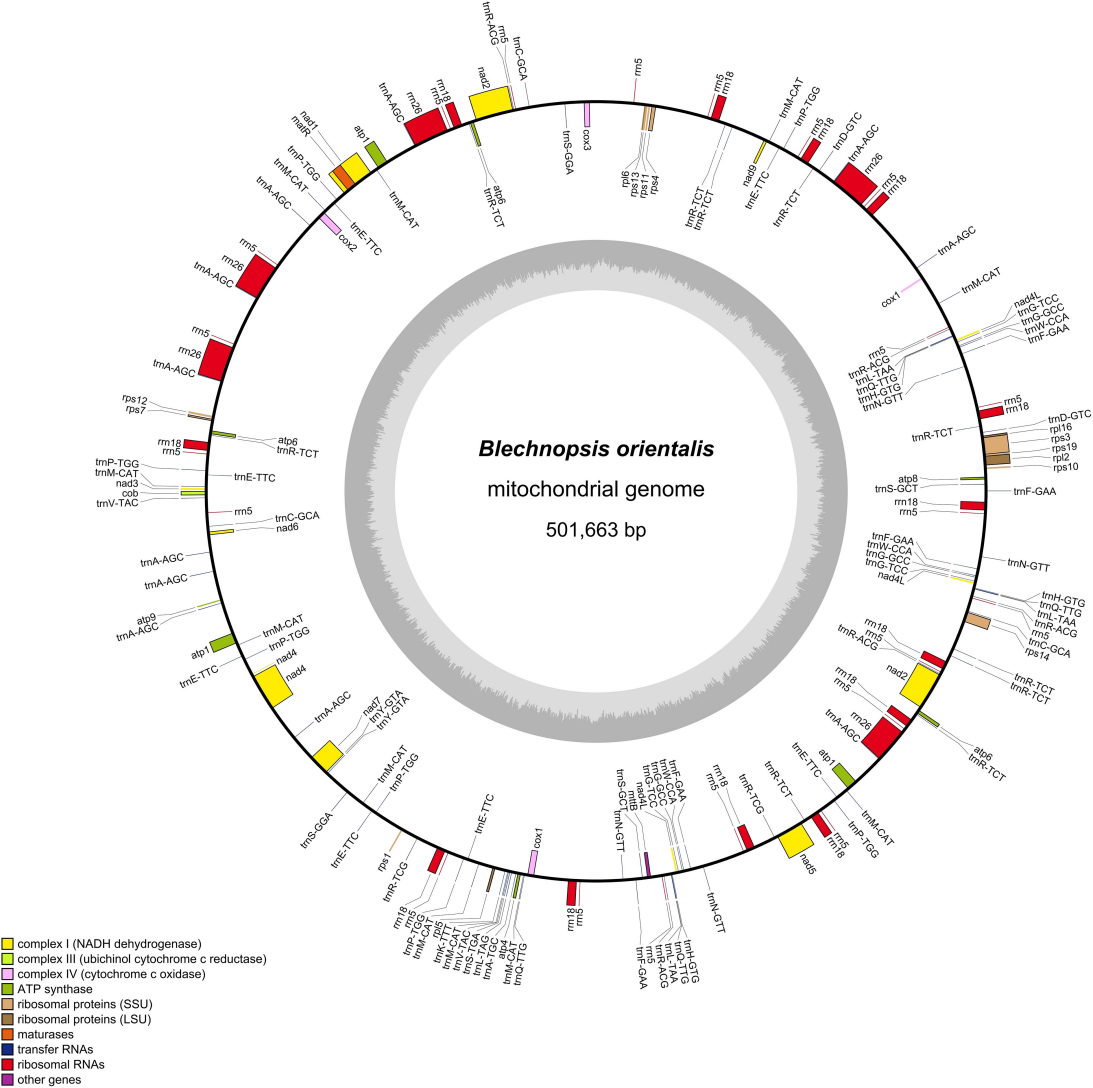
Circular maps of the *B*. *orientalis* mitochondrial. Genomic features mapped on the inside and outside of the circle. Colors were applied for different functional groups.

The mitochondrial genome of *B. orientalis* has a total length of 501,663 bp, with a GC content of 48.53%. Sequencing depth analysis reveals an average coverage of 239.19 × for second-generation sequencing and 54.82 × for third-generation sequencing (**Supplementary Figures S1**, **S2**). No sequence gaps were observed at any position, indicating high data quality. A total of 179 genes were annotated (**Figure 2**, **Table 1**), comprising 40 PCGs, 98 tRNA genes, 40 rRNA genes, and 1 pseudogene (*rps11*).

**Table 1:** The genes of *B. orientalis* mitochondrial genome.

Group of genes	Name of genes
ATP synthase	atp1*(3), *atp4*, *atp6*(3), *atp8*, *atp9*
Ubichinol cytochrome c reductase	*cob*
Cytochrome c oxidase	cox1*, cox2*, *cox3*
Maturases	matR*
Transport membrance protein	*mttB*
NADH dehydrogenase	nad1***, nad2****(2), *nad3*, nad4***, *nad4L*(3), nad5***, *nad6*, nad7***, *nad9*
Ribosomal proteins (LSU)	*rpl16*, rpl2*, *rpl5*, *rpl6*
Ribosomal proteins (SSU)	#*rps11*, *rps1*, *rps10*, *rps12*, *rps13*, rps14*, *rps*19, rps3**, *rps4*, *rps7*
Ribosomal RNAs	*rrn18*(13), rrn26*(5), *rrn5*(22)
Transfer RNAs	trnA-AGC*(11), *trnA-TGC*, *trnC-GCA*(3), *trnD-GTC*(2), *trnE-TTC*(7), *trnF-GAA*(5), *trnG-GCC*(3), *trnG-TCC*(3), *trnH-GTG*(3), *trnK-TTT*, *trnL-TAA*(3), *trnL-TAG*, *trnM-CAT*(11), *trnN-GTT*(4), *trnP-TGG*(7), *trnQ-TTG*(4), *trnR-ACG*(5), *trnR-TCG*(2), *trn*R*-TCT*(10), *trnS-GCT*(2), *trnS-GGA*(2), *trnS-TGA*, *trnV-TAC*(2), *trnW-CCA*(3), *trnY-GTA*(2)

*: intron number; #Gene: Pseudo gene; Gene (2): Number of copies of multi-copy genes.

### 3.2 Comparison of mitochondrial genome

**Figure 3:**
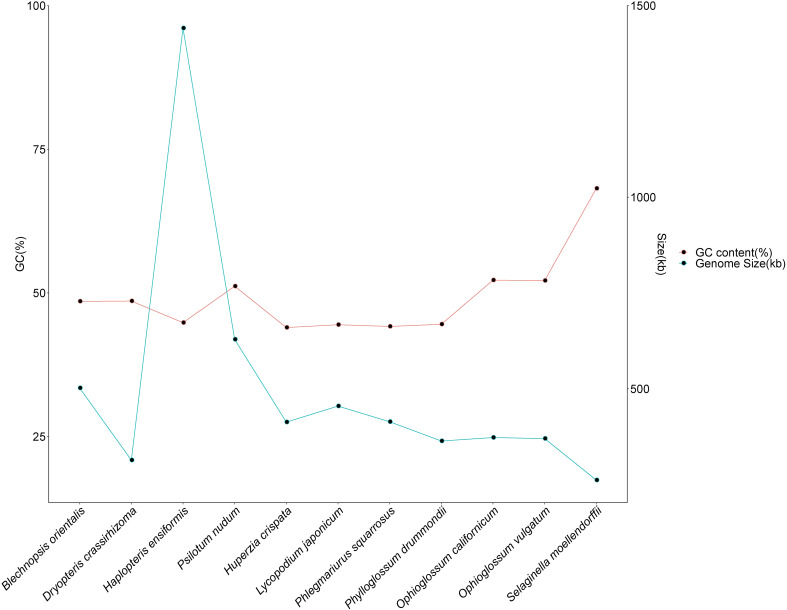
Comparison of GC content and genome size of *B. orientalis* mitochondrial genome with other ten fern and lycophyte species.

To examine the evolutionary characteristics of *B. orientalis* mitochondrial genome, a comparative analysis was conducted with the mitochondrial genome of ten other fern and lycophyte species. The results (**Figure 3**) indicated that *B. orientalis* mitochondrial genome exhibited moderate GC content and genome size relative to the other ten species. The GC content of the six fern species ranged from 42.16% to 52.22%, while the five lycophyte species had GC content from 43.97% to 68.18%, showing minimal variation across families and genera. The smallest mitochondrial genome length was observed in *D. crassirhizoma* (313.364 kb), while the largest was found in *H. ensiformis* (1,441.248 kb). The mitochondrial genome sizes of the five lycophyte species ranged from 261.212 kb to 454.458 kb, exhibiting relatively limited variation in size.

**Figure 4:**
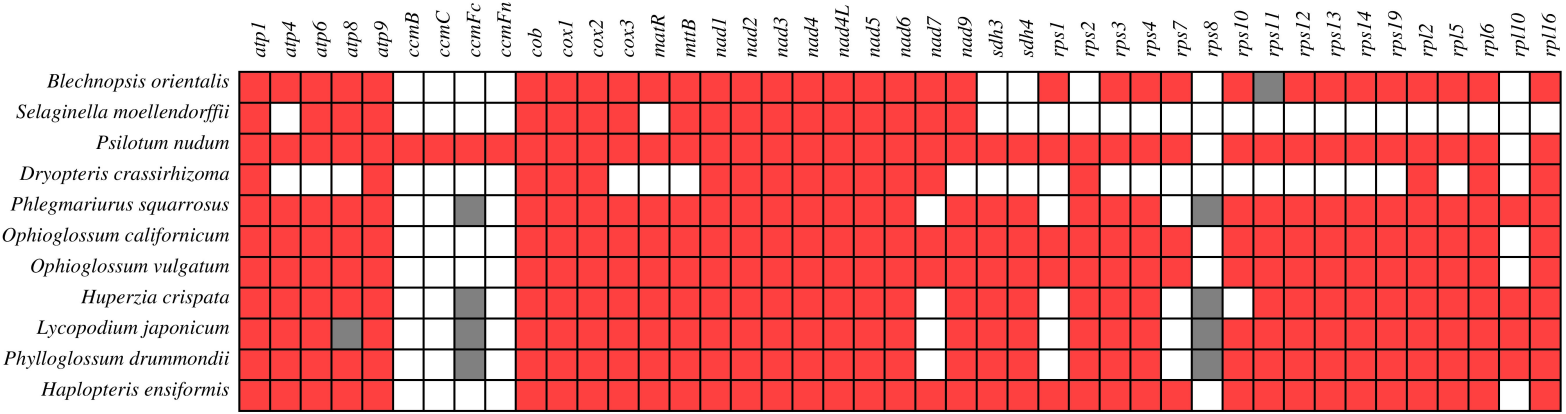
Presence and absence of mitochondrial genome PCGs of *B*. orientalis compared with ten other fern and lycophyte species. Red denotes the presence of a gene in the corresponding species, white indicates its absence, and gray signifies the presence of a pseudogene.

The 43 PCGs shared among mitochondrial genome of these 11 species were analyzed comparatively (**Figure 4**). In contrast to other ferns, *B. orientalis* exhibited distinctive features in gene loss or variation, notably the absence of *rps2* and the presence of a pseudogene (*rps11*) in its mitochondrial genome. Generally, ferns and lycophytes display divergent patterns of gene loss. For instance, all fern species lacked *rps*10, while lycophytes demonstrated a tendency to lose *rps1* and *rps7*. Furthermore, four species within the Lycopodiaceae family contained pseudogenes *ccmFn* and *rps8*. Certain genes, including *atp1*, *atp4*, *atp6*, *cox1*, and *nad1*, were present in nearly all species, whereas genes such as *rps3*, *rps12*, *rps13*, and *rps14* exhibited varying degrees of loss across different species, underscoring species-specific differences in their absence. The *ccm* gene series was identified only in *P. nudum*, while the other ten species either lacked or possessed pseudogenes for these genes. The *rps8* gene was either absent or present as a pseudogene in all 11 species examined.

### 3.3 Ka/Ks analysis of mitochondrial genome PCGs

**Figure 5:**
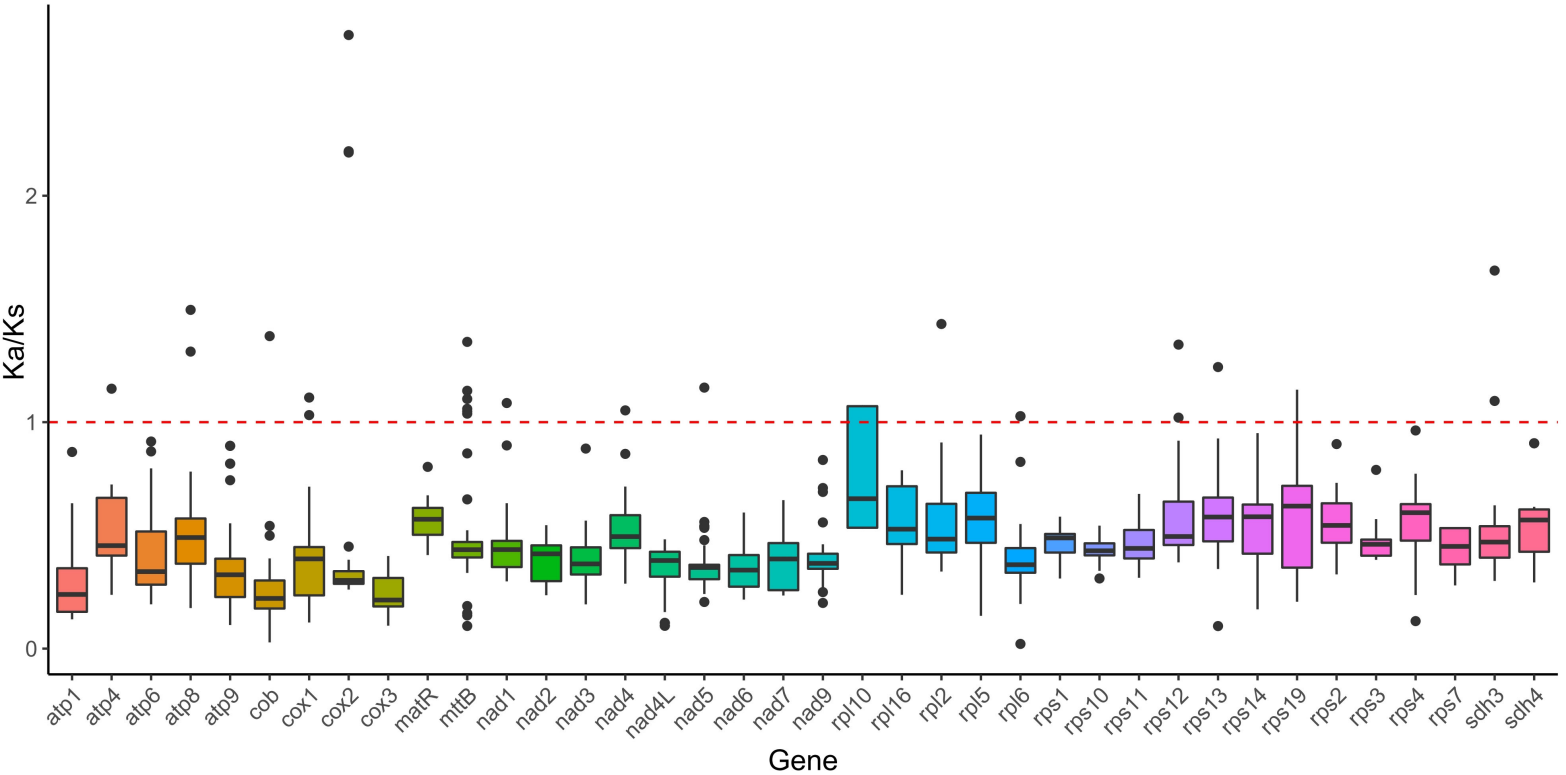
Ka/Ks analysis of mitochondrial genome PCGs of *B*. *orientalis* compared with ten other fern and lycophyte species. The solid black line on the box plot depicts the mean value, while the dotted red line indicates the median.

Various species encounter different ecological pressures during evolution, which subsequently result in alterations in their genomes. The Ka/Ks ratio serves as a measure of selection pressure on PCGs during evolution, enabling inferences about species adaptation and evolutionary trajectories. To evaluate the selection pressure acting on the PCGs of *B. orientalis* and ten additional fern and lycophyte species, we calculated Ka/Ks values for 38 mitochondrial genome PCGs (**Figure 5**). The average Ka/Ks value < 1 for all PCGs in the *B. orientalis* mitochondrial genome indicates that these 38 PCGs have undergone purifying selection throughout evolution, maintaining relatively stable protein functions. The Ka/Ks analysis further demonstrated that all PCGs in the mitochondrial genome of these 11 plant species exhibited negative selection and were highly conserved during their evolutionary history.

### 3.4 Codon preference analysis

**Figure 6:**
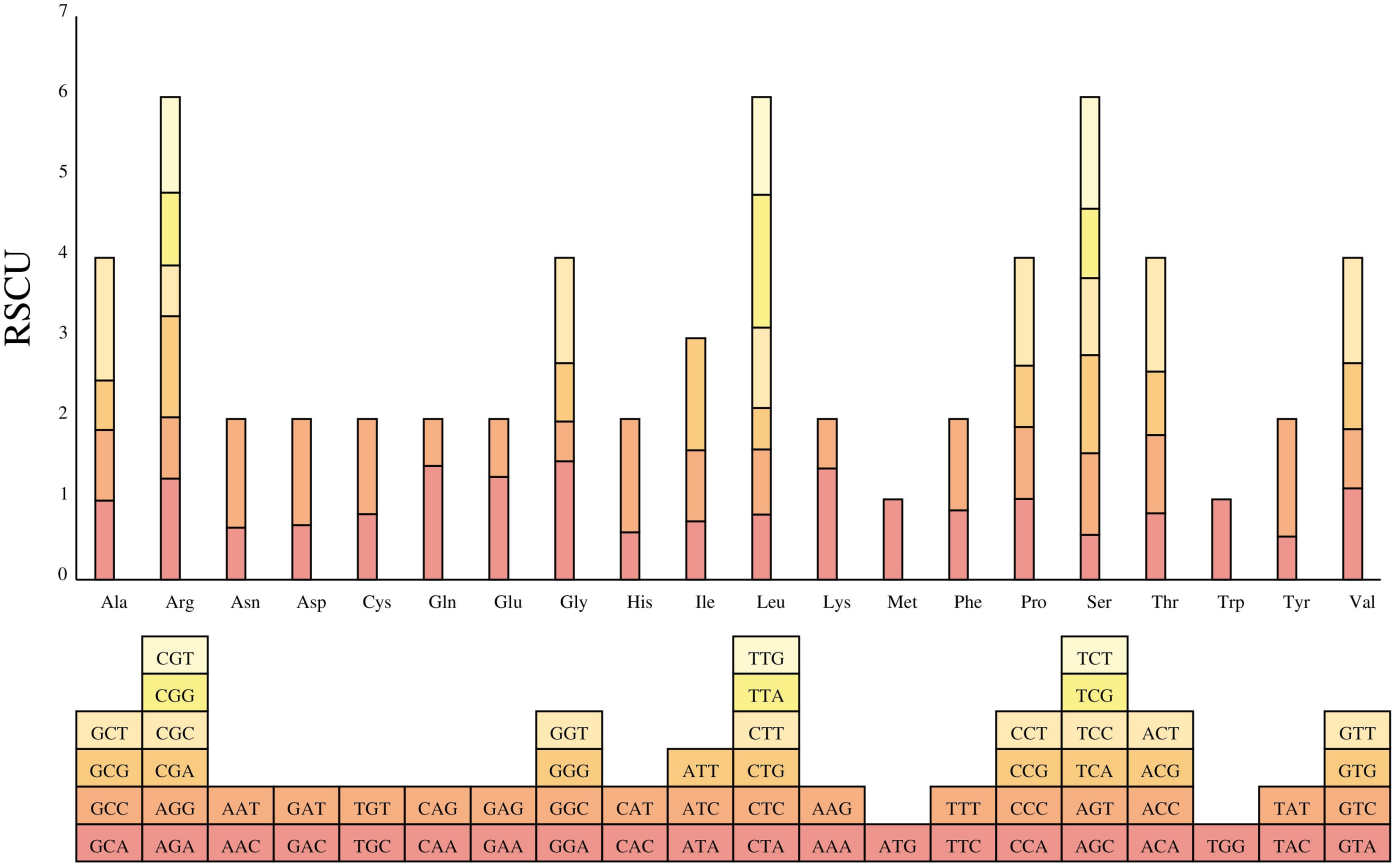
Relative synonymous codon usage from *B*. *orientalis* mitochondrial genome. The histogram’s color corresponds to that of the codon.

The codon usage preference of PCGs in *B. orientalis* mitochondrial genome was analyzed. A codon is considered preferentially used by amino acids when its RSCU value > 1. The analysis (**Figure 6**) identified 9,125 codons in the mitochondrial genome PCGs of *B. orientalis*, revealing general codon preferences, particularly for amino acids with multiple synonymous codons, except for the start codon (AUG) and tryptophan (UGG) (RSCU = 1). The PCGs of *B. orientalis* mitochondrial genome contained 5,081 high-frequency codons (RSCU > 1), predominantly ending in A or T bases, indicating a preference for such codons. Among these high-frequency codons, the third position was generally either A or U, with the exception of UUG. For low-frequency codons (RSCU < 1), the third position was predominantly G or C. This feature is characteristic of codon bias in organelle genomes of terrestrial plants. Notably, leucine (Leu) exhibited a preference for the UUA codon, with the highest RSCU value among mitochondrial PCGs, reaching 1.6484.

**Figure 7:**
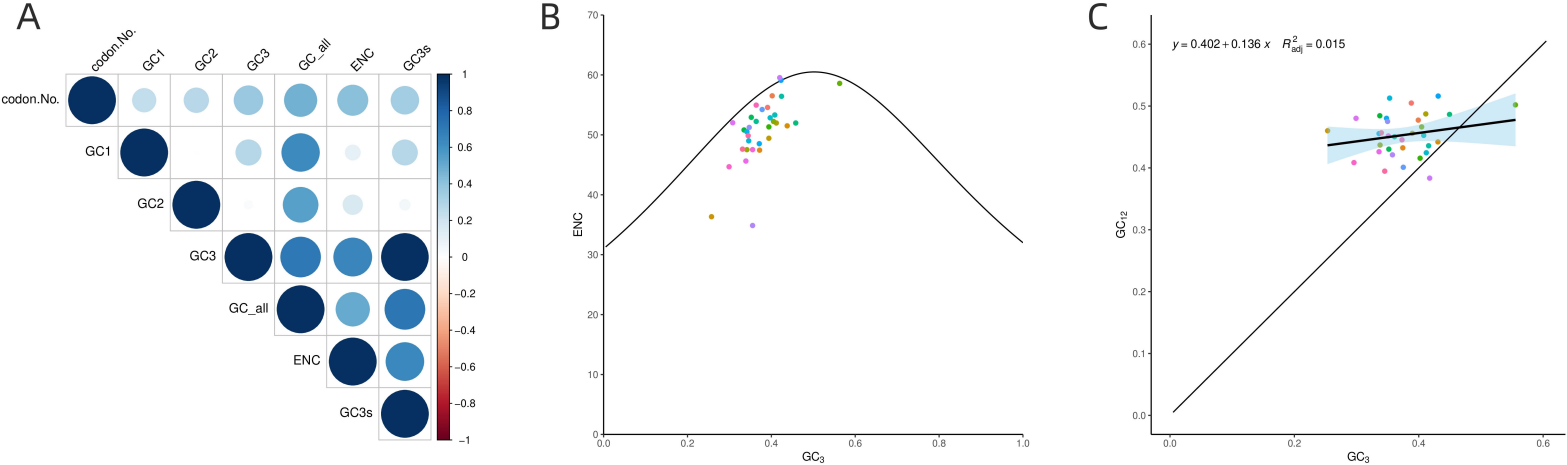
The analysis of GC content at different positions in PCGs **(A)**, ENC-GC3 correlation **(B)**, and the neutrality plot **(C)**. **(A)** The size of the circles represents the variation in GC content at different positions, with varying colors indicating the magnitude of the values. **(B)** The standard curve is calculated using the equation: ENC=2+GC3 + 29/[GC3²+(1-GC3)²]. **(C)** Individual genes are represented by different colored points, while the black line illustrates the overall trend.

Analysis of GC content at different positions within PCGs (**Figure 7A**) revealed that the GC content at the GC1 in PCGs of *B. orientalis* mitochondrial genome ranged from 37.16% to 57.12%, with an average of 47.55%. The GC content at GC2 ranged from 30.1% to 52.13%, averaging 43.07%, while at GC3 it ranged from 25.33% to 55.56%, averaging 37.55%. The GCall varied from 37.09% to 51.98%, with an average of 42.73%. These findings suggest that cytosine (C) and guanine (G) are more prevalent at GC2. Correlation analysis indicated that GC1 was negatively correlated with GC2 but positively correlated with GC3 and ENC. Moreover, GC1 demonstrated a highly significant positive correlation with GCall (P < 0.001). GC2 positively correlated with GC3 and ENC, and exhibited a highly significant positive correlation with GCall (P < 0.001). Similarly, GC3 showed a highly significant positive correlation with both GCall and ENC (P < 0.001). Additionally, GCall displayed a significant positive correlation with ENC (P < 0.01).

The ENC-GC3 correlation analysis (**Figure 7B**) reveals a broad distribution of *B. orientalis* mitochondrial DNA. The majority of genes are positioned below the standard curve, with a substantial distance from it, indicating that natural selection primarily influences codon usage bias for most genes. However, a small number of genes are located slightly above the standard curve, suggesting that mutational pressure has a greater influence on their codon usage bias. The analysis shows that 61% of the genes had ENC ratio values between 0.05 and 0.15 (20 genes), while 9 genes had values between -0.05 and 0.05. This distribution indicates that the actual ENC values of most genes deviate considerably from the theoretical ENC values, suggesting that codon usage bias in *B. orientalis* is subject to limited mutational pressure but is strongly influenced by natural selection. In conclusion, both mutational and natural selection pressures influence codon usage bias in *B. orientalis* mitochondrial genome, with natural selection exerting a more dominant effect.

The neutral plot (**Figure 7C**) illustrates that the GC content at GC12 ranges from 0.384 to 0.516, while the GC content at GC3 spans from 0.253 to 0.556. The low regression slope (0.136) indicates a weak correlation between GC3 and GC12, suggesting that mutations have differential effects on the GC content at the first, second, and third codon positions. These observations imply that base mutations exert a limited influence on the codon preference of *B. orientalis* genes.

### 3.5 Repeat sequence analysis

**Figure 8:**
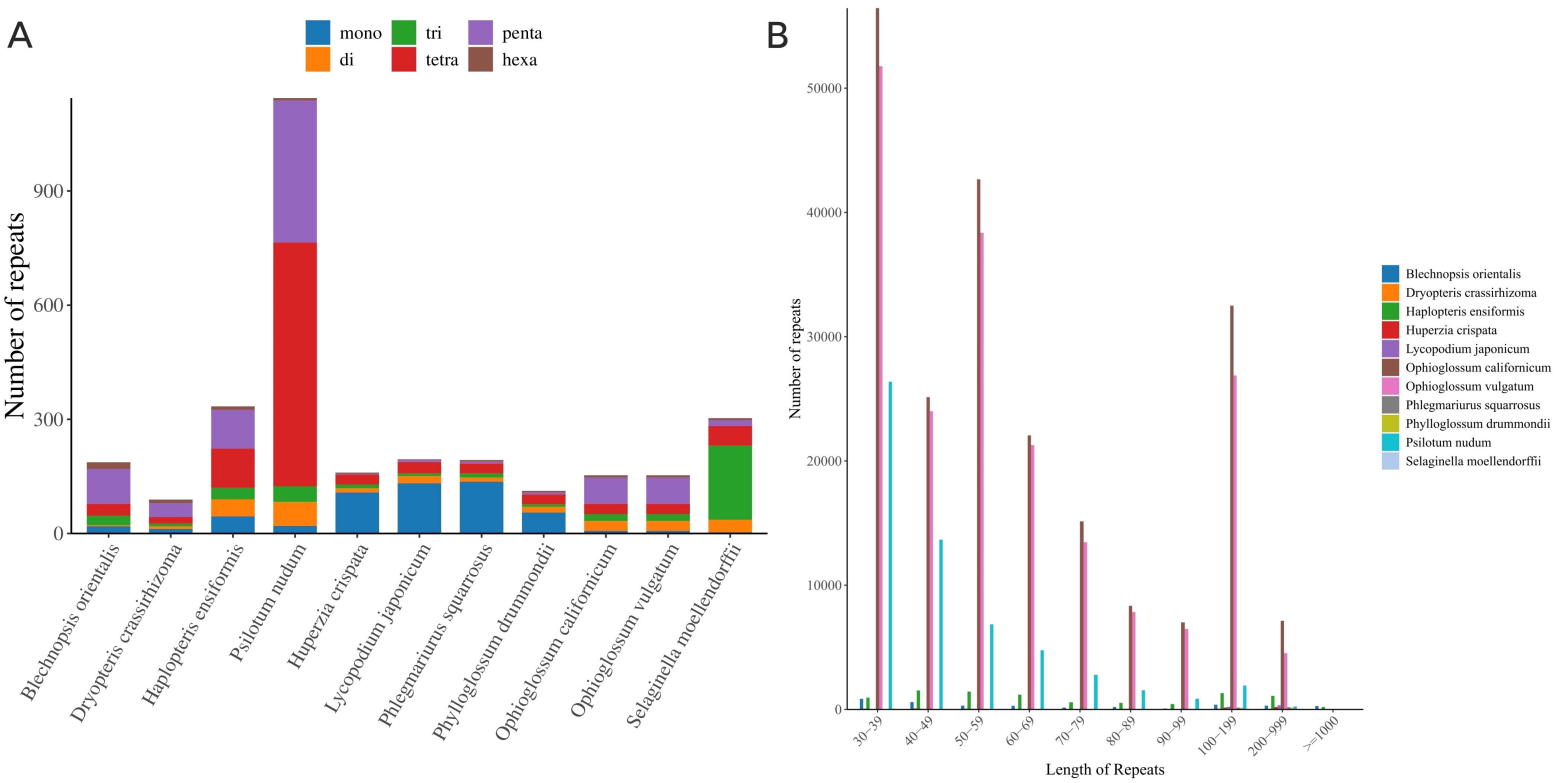
Comparative analysis of repeat sequences in mitochondrial PCGs of *B*. *orientalis* with ten other fern and lycophyte species. **(A)** the type and number of SSRs detected. **(B)** the number of long repeat sequences.

Simple sequence repeat (SSR) analysis (**Figure 8A**) identified 187 SSRs in *B. orientalis* mitochondrial genome. Pentanucleotide repeats were the most prevalent, comprising 92 instances and accounting for 49.20% of the total. These repeats primarily occurred in the short length range of 30-39 bp, with no amplification events involving long repeats. A comparative analysis among 11 fern and lycophyte species revealed significant variations in both the quantity and types of repeat sequences. Lycophytes exhibited relatively few repeat sequences, predominantly simple mononucleotide and dinucleotide repeats. In contrast, ferns displayed a higher number of repeats, particularly in the genera *P. nudum* and *Ophioglossum*. Notably, *P. nudum* contained 1147 SSRs, while the remaining 10 species had between 89 and 334 SSRs. In the mitochondrial genome of *L. japonicum*, *P. squarrosus*, and *P. drummondii*, mononucleotide repeats were predominant, whereas the other eight species exhibited a prevalence of tetranucleotide and pentanucleotide repeats, or a relatively balanced distribution of repeat types. The number of tetranucleotide and pentanucleotide repeats in *P. nudum* significantly exceeded that of other nucleotide types.

The analysis and comparison of repeat number and length relationships across 11 species revealed a prevalence of short repeats (30-49 bp) in all species (**Figure 8B**), particularly in *S. moellendorffii* and *P. nudum*, indicating their widespread presence in these genomes. Medium-length repeats (100-199 bp) were more common in *P. nudum* and *O. californicum*, potentially suggesting specific biological functions or associations with particular evolutionary events in these species. Lycophytes exhibited a more concentrated distribution of repeats compared to ferns, with short repeats predominating, especially in *L. japonicum* and *H. crispata*. The broader distribution of repeat lengths in ferns, such as *P. nudum*, demonstrated a substantial presence of short to medium-length repeats, possibly reflecting extensive expansion or duplication events during genome evolution. Other ferns, including *O. californicum* and genus *O. vulgatum*, also displayed significant distributions of longer repeat sequences.

### 3.6 Analysis of RNA editing sites

**Figure 9:**
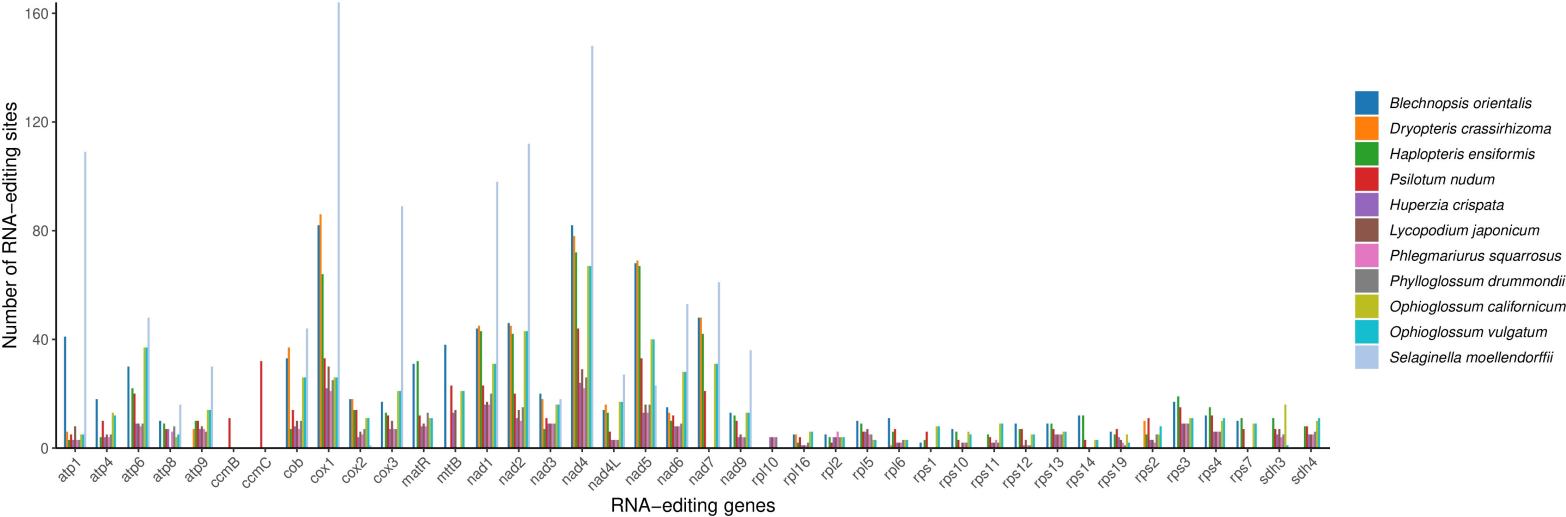
Comparative analysis of RNA editing sites in *B*. *orientalis* and ten other fern and lycophyte species. Distinct colors are used to represent different species.

RNA editing sites were examined across 43 PCGs in 11 fern and lycophyte species (**Figure 9**), revealing 783 RNA editing events in the mitochondrial genome of *B. orientalis*, indicating a high frequency of editing sites. These events primarily comprised cytosine to uracil (C-to-U) and uracil to cytosine (U-to-C) conversions. RNA editing was predominantly concentrated in the *atp1* gene and primarily involved transformations of hydrophobic amino acids. The most frequent alterations were serine (S) to leucine (L) and serine (S) to phenylalanine (F), accompanied by a substantial number of proline (P) to leucine (L) and proline (P) to phenylalanine (F) conversions. These extensive modifications in hydrophobic amino acids may influence protein structural stability and membrane integration, potentially modulating the function of mitochondrial proteins.

Among the species analyzed, *S. moellendorffii* displayed the highest number of RNA editing sites, totaling 1,077, predominantly concentrated in genes such as *cox*1 and *nad*5. This species exhibited significantly more editing sites than any other, while *P. squarrosus* demonstrated the least. The majority of genes, particularly those in the *atp* and *rps* series, showed very few RNA editing events, with some cases having almost none. Generally, lycophytes (excluding *S. moellendorffii*) presented fewer RNA editing sites compared to the 11 species examined, whereas ferns exhibited higher RNA editing activity, especially in genes associated with mitochondrial respiration and energy metabolism. With the exception of *S. moellendorffii*, most lycophytes displayed relatively few RNA editing sites, particularly in genes like *atp* and *rps*. However, a few key genes, such as *cox*1, *nad4*, and *nad5*, still showed a higher number of editing events.

### 3.7 Collinearity analysis

**Figure 10:**
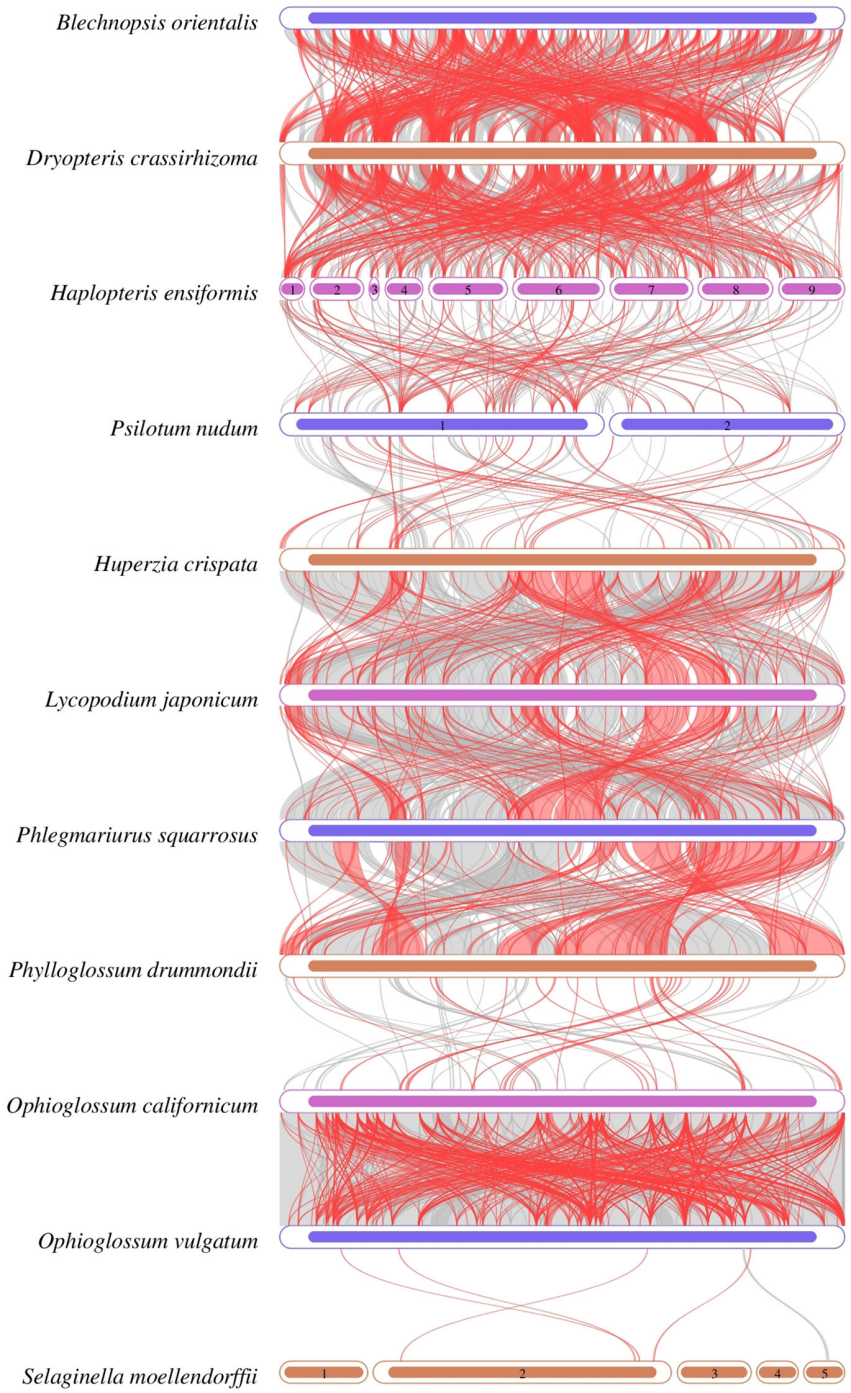
Collinearity analysis of *B*. *orientalis* with ten fern other and lycophyte species. Regions connected by arcs indicate areas with significant homology. Red arcs represent sequence reversals, while gray regions denote positive sequences. Regions lacking collinear blocks suggest species-specific unique sequences.

The collinear regions of *B. orientalis* mitochondrial genome and ten other species of ferns and lycophytes were analyzed (**Figure 10**). The analysis revealed that *B. orientalis* and the other species share numerous homologous collinear genome blocks. The collinear lines between *B. orientalis* and *D. crassirhizoma* were particularly dense, with the total length of collinear blocks reaching 292,034 bp, constituting 58.216% of the mitochondrial genome length of *B. orientalis*. In contrast, the collinearity between *B. orientalis* and lycophytes was comparatively weak, with *S. moellendorffii* exhibiting almost no collinearity. Furthermore, the arrangement of collinear blocks in each mitochondrial genome varied, indicating that *B. orientalis* has undergone substantial genome rearrangements compared to related species. These findings suggest that *B. orientalis* mitochondrial genome exhibits a high degree of structural variability.

### 3.8 Identification of homologous fragment

**Figure 11:**
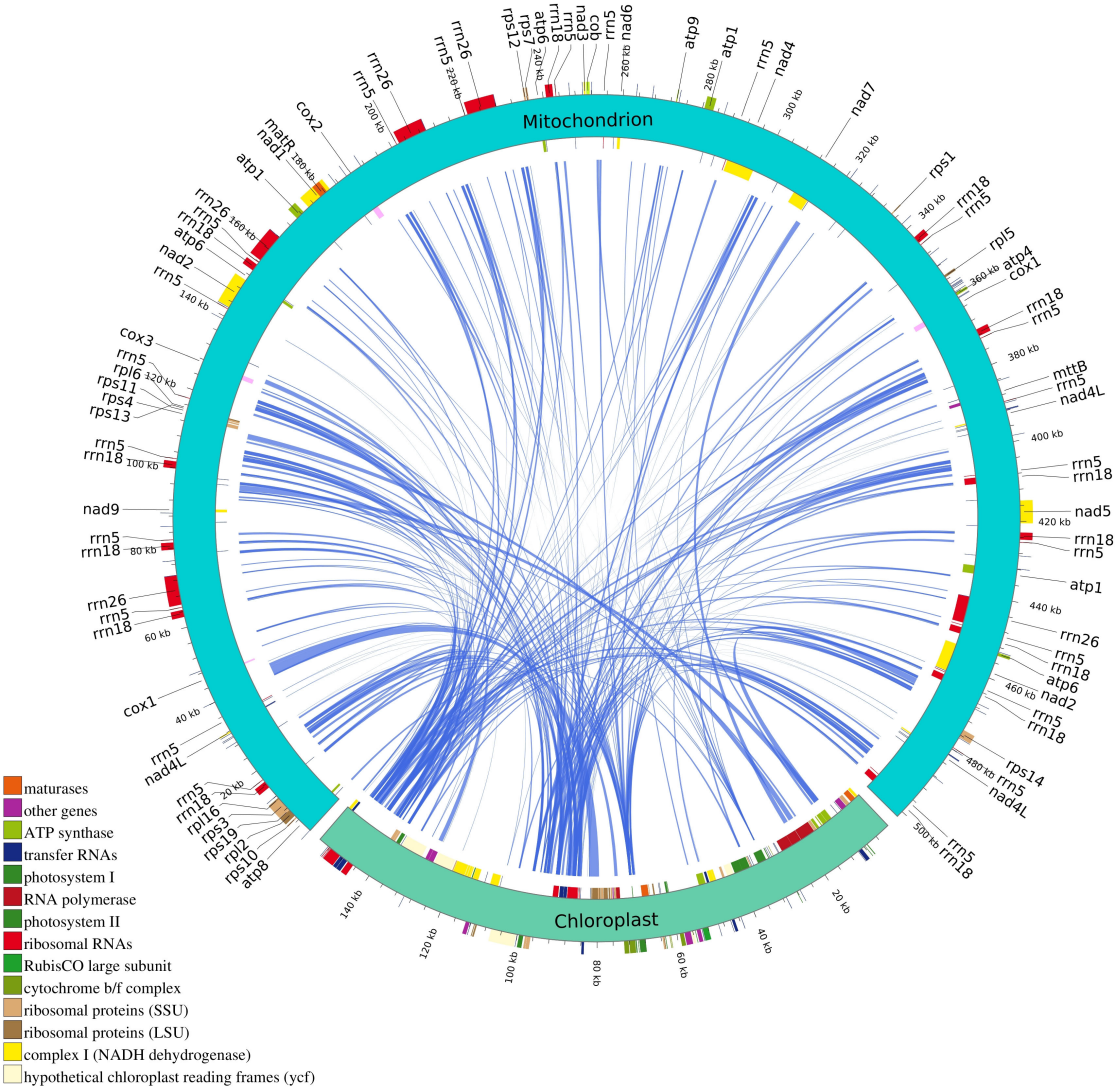
Transfer events of chloroplast genome from *B. orientalis* to mitochondrial genome. The turquoise arc represents the mitochondrial genome. The light green arc depicts the chloroplast genome. The blue inner arc illustrates the corresponding transfer mitochondrial plastid DNA sequence (MTPT).

Intracellular genetic material transfer is a common phenomenon in mitochondrial genome during the evolution of higher plants. In the mitochondrial genome of *B. orientalis*, 284 sequence fragments were identified based on nucleotide sequence similarity, with lengths ranging from 48 to 2,951 bp. The longest homologous fragment, measuring 2,951 bp, was located at positions 39,545-42,495 bp of the mitochondrial genome. Homologous mitochondrial fragments totaled 74,719 bp, accounting for 14.89% of the mitochondrial genome, while homologous chloroplast fragments totaled 34,194 bp, representing 22.03% of the chloroplast genome (**Figure 11**). Annotation of these homologous sequences revealed 10 complete genes, including 5 PCGs (*rps19*, *rp*l*2*, *rpl23*, *psbN, psbH*), four tRNA genes (*trnI-CAU*, *trnR-UCU*, *trnR-ACG*, *trnM-CAU*), and one rRNA gene (*rrn4.5*). Additionally, 25 partial genes were identified, including 18 PCGs (*atpA*, *atpE*, *atpF*, *chlN*, *ndhB*, *ndhH*, *psaB*, *psbA*, *psbB*, *psbC*, *rbcL*, *rpl22*, *rpoA*, *rpoC1*, *rpoC2*, *rps11*, *rps12*, *ycf2*), four tRNA genes (*trnA-UGC*, *trnG-UCC*, *trnI-GAU*, *trnR-ACG*), and three rRNA genes (*rrn5*, *rrn16*, *rrn23*). Furthermore, some small chloroplast-derived fragments were found to be subsets of larger sequences or occurred multiple times in the mitochondrial genome, suggesting that these fragments may have undergone multiple independent transfers, replications, and recombination events within the mitochondrial genome after their integration.

### 3.9 Phylogenetic analysis

**Figure 12:**
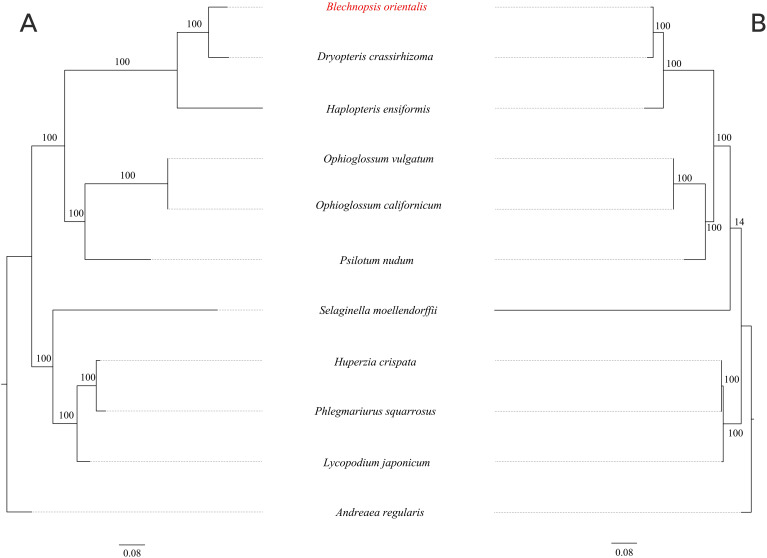
The ML phylogenetic tree of chloroplast genome and mitochondrial genome of *B*. *orientalis.* **(A)** chloroplast genome phylogenetic tree. **(B)** mitochondrial genome phylogenetic tree.

**Figure 13:**
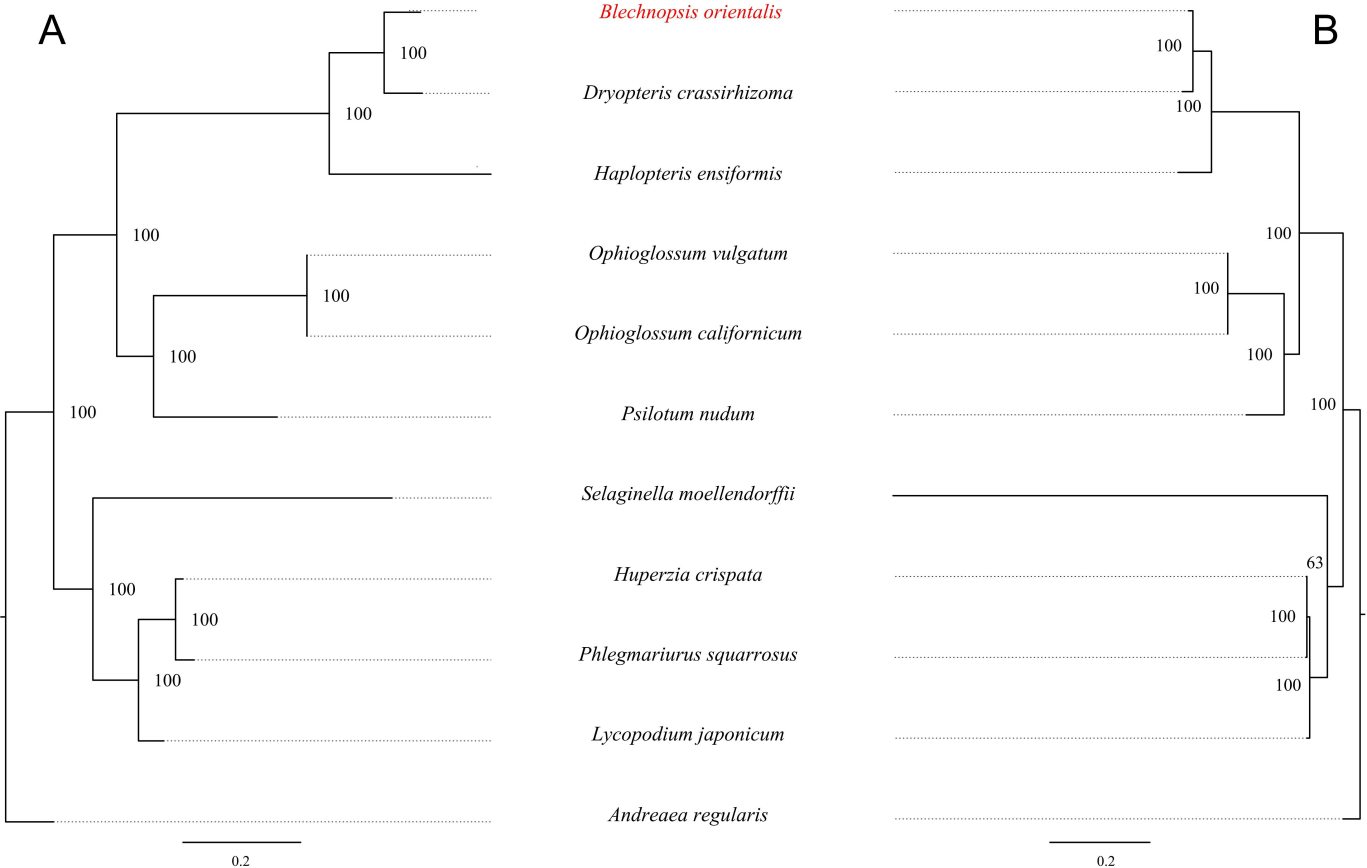
The BI Phylogenetic tree of chloroplast genome and mitochondrial genome of *B*. *orientalis.* **(A)** chloroplast genome phylogenetic tree. **(B)** mitochondrial genome phylogenetic tree.

To investigate the evolutionary relationship of *B. orientalis*, we used *A. regulari*s as the outgroup and employed both ML and BI methods to analyze the phylogenetic relationships among *B. orientalis* and nine other fern and lycophyte species. Phylogenetic trees were constructed using 31 mitochondrial PCGs and 83 chloroplast PCGs from the selected species (**Figures 12**, **13**). The results indicated that the trees generated by the two methods exhibited similar topologies, particularly for the phylogenetic trees constructed based on the chloroplast genome, where the major clades and branching orders were largely consistent between the two analyses. Both tree-building methods, as well as the analyses based on the two types of genes, effectively distinguished *B. orientalis* from the other fern and lycophyte species. *B. orientalis* was placed within the ferns and was more closely related to the other four fern species, with *B. orientalis* and *D. crassirhizoma* clustering together, indicating a close evolutionary relationship.

## 4 Discussion

### 4.1 Structure and characteristics of mitochondrial genome

The mitochondrial genome in higher plants often exhibits complex circular or linear structures due to the presence of large repeat sequences (Smith and Keeling, 2015). Structural variations of mitochondrial genome have been documented across seed plants, ferns, and lycophytes. For instance, *A. biserrata* mitochondrial genome comprises six circular chromosomes (Wang L. et al., 2024), while *Pteridium revolutum* possesses 13 linear chromosomes (Feng and Wicke, 2022), and *Selaginella moellendorffii* contains five linear chromosomes (Hecht et al., 2011). The mitochondrial genome of *B. orientalis* is a complex conformation of a circular chromosome. The *rps11* gene typically encodes ribosomal protein S11, which functions in mitochondrial or cytoplasmic ribosomes and plays a crucial role in maintaining normal cellular metabolism and growth. In *B. orientalis* mitochondrial genome, *rps11* is identified as a pseudogene. This may result from the loss of gene function caused by long-term evolutionary processes under selection pressure or genetic mutation. The conversion of *rps11* into a pseudogene or its complete deletion is relatively common in plant mitochondrial studies, as also observed in *Photinia serratifolia* (Wang et al., 2023).

### 4.2 Comparison of the *B. orientalis* mitochondrial genome

The size of plant mitochondrial genomes is closely associated with the abundance of non-coding sequences and repeat elements (Mower et al., 2012). The relatively large size of *B. orientalis* mitochondrial genome can be attributed to the extensive presence of non-coding sequences. Analysis of repeat sequences indicates that, unlike other plant species, significant long repeat amplification events are absent in *B. orientalis* mitochondrial genome. With a GC content of approximately 50%, *B. orientalis* appears to have maintained balanced genomic stability and mutation rates throughout its evolutionary history. Previous studies have indicated that GC content is significantly associated with gene expression regulation and adaptive evolution (Qiao et al., 2022). Moderate GC content contributes to genomic stability and enhances adaptability under specific environmental conditions (Li et al., 2023). The analysis also revealed that the GC content of ferns is relatively stable compared to that of lycophytes, with smaller fluctuations among different families and genera. In contrast, lycophytes exhibit more extreme GC content values, such as *S. moellendorffii* mitochondrial genome, which has a GC content as high as 68.18%, consistent with previous findings (Kang et al., 2020). Compared to lycophytes, the size of fern mitochondrial genome shows greater variation across different families and genera. In some fern species, the mitochondrial genome size exceeds 1,000 kb, such as *H. ensiformis*, which has an mitochondrial genome size of 1,441.248 kb (Zumkeller et al., 2023).

Analysis of gene loss in mitochondrial genome PCGs across these plant species reveals that *B. orientalis* exhibits the absence or variation of certain genes, such as *rps8* and *rps10*. These genes loss may be associated with early nuclear/cytoplasmic-origin gene loss and replacement events during the evolutionary history of *B. orientalis*, consistent with findings reported in other plant studies (Adams et al., 2002; Murcha et al., 2005). In this investigation, all examined plants except *P. nudum* demonstrated gene loss or pseudogenization in the *ccm* gene series, further corroborating previous research indicating that *ccm* genes are frequently absent or exist as pseudogenes in lycophytes and some ferns (Sun N, et al., 2024). It is hypothesized that during the evolution of *B. orientalis*, gene transfer to the nuclear genome may have resulted in the loss of *ccm* genes in its mitochondrial DNA.

Furthermore, significant variations were observed in mitochondrial genome PCGs among lycophyte species, particularly those within the Lycopodiaceae family, and *S. moellendorffii*. These distinctions primarily involve gene loss and pseudogenization in the *ccm* and *rps* gene series. Notably, *S. moellendorffii* displays patterns in *ccm* and *rps* genes that more closely resemble those of ferns, especially in the case of *rps10*.

### 4.3 Ka/Ks analysis

Ka/Ks analysis serves as a valuable method for elucidating species adaptability, selective pressures, adaptive potential, and genetic and evolutionary processes (Xie et al., 2019). The Ka/Ks analysis of shared mitochondrial PCGs between *B. orientalis* and ten other fern and lycophyte species revealed that all selected species exhibited Ka/Ks values < 1. This finding indicates that these genes possess conserved protein functions and have been highly preserved throughout evolution. Previous studies on Ka/Ks values in lycophytes and ferns have demonstrated that most genes display Ka/Ks values < 1, suggesting that the majority are subject to negative selection pressure (Cao et al., 2023; Sun W, et al., 2024). The Ka/Ks analysis results for *B. orientalis* further corroborate that PCGs in these ancient plants, including ferns and lycophytes, have undergone strong evolutionary pressures, resulting in purifying selection to mitigate detrimental mutations and maintain their essential functions.

### 4.4 Codon preference analysis

Codon bias affects gene expression, translation efficiency, and genome evolution, with both natural selection and mutational biases influencing codon usage in various genes (Hershberg and Petrov, 2008). The RSCU value represents the ratio between actual and theoretical codon usage frequencies, proving valuable for assessing species-specific codon preferences. Analyzing codon bias is essential for comprehending species evolution (Sharp et al., 1986). The codon preference in *B. orientalis* mitochondrial PCGs is mainly seen in amino acids with degenerate codons, especially leucine, arginine, and serine, favoring A/T-ending synonymous codons with high consistency and low variability. Interestingly, this pattern contrasts with previous observations in lycophytes and ferns, which generally exhibit high variability in codon usage and a strong preference for codons ending in A/U (Xu et al., 2024). This observation unveils diverse adaptations in codon usage and genomic patterns among plants along different evolutionary trajectories.

Codon preference analysis revealed a broad distribution in *B. orientalis* mitochondrial DNA, with codon preferences primarily driven by natural selection and minimal influence from mutations. These findings are consistent with previous studies on fern and lycophyte species, including *Ceratopteris richardii* and *Adiantum capillus*-*veneris*, where codon bias is significantly influenced by both mutations and natural selection, with a stronger impact from the latter (Xu et al., 2024). Similar codon preference characteristics have been observed in *Equisetum hyemale* and other lycophytes (Kumar et al., 2022). Collectively, these studies indicate that codon usage preference in ferns and lycophytes is predominantly shaped by natural selection, with relatively low mutational pressure. This conclusion aligns with the findings for *B. orientalis*, further supporting the crucial role of natural selection in determining codon usage patterns in these ancient plant lineages.

### 4.5 Repeat sequence analysis

Plant mitochondrial genome contains numerous repetitive sequences, which play a crucial role in their evolution and significantly promote gene recombination within mitochondrial genome (Jiang et al., 2023; Cole et al., 2018; Li et al., 2022). Analysis showed that pentanucleotide repeats were the most common type in the *B. orientalis* mitochondrial genome, making up 49.20% of all repeats. This finding contrasts with previous studies on ferns and lycophytes, which reported a predominance of single-nucleotide and dinucleotide repeats in most plants (Sun et al., 2024). These results underscore the diversity in repeat sequence distribution among different plant species. In *B. orientalis*, repeats are primarily concentrated within the short length range of 30-39 bp, and no significant long repeat amplification events were observed, a characteristic more commonly seen in other ferns and lycophytes (Cao et al., 2023). This suggests that *B. orientalis* mitochondrial genome may have undergone limited substantial rearrangement events during evolution. Among the selected species, notable differences were observed in the number of repeat sequences and the dominant nucleotide types in the mitochondrial genomes of ferns and lycophytes. Ferns generally exhibit a higher number of repeats, with some species possessing more than one thousand SSRs, while lycophytes tend to have fewer repeat sequences. This highlights the variability in the abundance of mitochondrial genome repeat sequences between fern and lycophyte species.

### 4.6 Analysis of RNA editing sites

RNA editing, a post-transcriptional process occurring in all higher plants, contributes to the conservation of amino acid sequences in essential mitochondrial proteins (Wu et al., 2015; Handa, 2003). This process involves changes in nucleotide sequences, including insertions, deletions, and substitutions, resulting in alterations of genetic information, and is widespread among living organisms (Sun et al., 2016). In *B. orientalis* mitochondrial genome, RNA editing primarily encompasses C-to-U and U-to-C conversions. These editing events are predominantly concentrated in the *atp1* gene and are mainly associated with the conversion of hydrophilic amino acids (e.g., Ser, Pro) to hydrophobic amino acids (e.g., Leu, Phe, and Val). Such modifications likely influence protein structural stability and membrane insertion, thereby regulating mitochondrial protein function. Notably, unlike the RNA editing events typically observed in seed plant genomes, which predominantly involve C-to-U conversions, *B. orientalis* mitochondrial genome exhibits a large number of U-to-C conversions. This type of conversion is relatively uncommon and primarily occurs in ferns and lycophytes (Zhang et al., 2022). As a member of Blechnaceae family, *B. orientalis* aligns well with these previous research findings. In these plants, U-to-C conversion, as an RNA editing event, may have significant adaptive and evolutionary significance. For example, it alters the amino acid composition of proteins, regulates gene function, and affects mitochondrial function, thereby providing plants with more adaptive options to survive and reproduce in complex or extreme environments.

Examination of RNA editing sites in 43 PCGs across 11 species of ferns and lycophytes revealed that ferns generally demonstrate elevated RNA editing activity, particularly in genes related to the respiratory chain. However, the quantity of RNA editing sites varies considerably among species. For example, *B. orientalis* exhibits a substantially higher number of editing sites compared to other species, while genus *Ophioglossum* species are more conservative in this aspect, suggesting diversity in gene regulation mechanisms.

### 4.7 Collinear analysis

Collinearity analysis serves as a valuable tool for investigating evolutionary relationships between species by examining the associations and sequence similarities among homologous genes (Xu et al., 2023). The extent of collinearity between two species can function as an indicator of evolutionary distance, offering insights into phylogenetic relationships. The rearrangement of plant mitochondrial genomes plays a crucial role in promoting genetic diversity, driving adaptive evolution, and influencing plant development and reproductive processes. The mitochondrial genome of *B. orientalis* has undergone substantial genomic rearrangements compared to closely related species, indicating a highly non-conservative structure. Nevertheless, *B. orientalis* and *D. crassirhizoma* display relatively high levels of syntenic blocks, suggesting a significant degree of homology. This implies that the two species may share numerous ancestral genes, pointing to a close evolutionary relationship. As a fern, *B. orientalis* also exhibits more syntenic regions with other ferns compared to lycophytes. This observation likely relates to the extensive genomic rearrangements and mutations that occurred in lycophytes during their evolution (Yu et al., 2023), resulting in fewer conserved regions shared with ferns.

### 4.8 Identification of homologous fragment

During the evolution of higher plants, mitochondrial genome frequently incorporates sequences derived from plastid DNA, known as MTPTs (Gui et al., 2016). This study reveals that *B. orientalis* mitochondrial genome contains numerous transferred segments from the chloroplast genome, demonstrating gene flow from chloroplast genome to mitochondrial genome. These transferred fragments encompass both complete and partial genes, suggesting potential integration of chloroplast genome functions into mitochondrial genome. The repeated duplication and recombination of these fragments indicate a complex dynamic process within the genome. Many fern species exhibit high recombination frequencies between chloroplast genome and mitochondrial genome, associated with genome expansion, particularly involving increases in non-coding regions and chloroplast gene transfers to the mitochondrial genome (Liu et al., 2012b). The mitochondrial genome of the lycophyte *H. squarrosa* also demonstrates a gene transfer mechanism similar to that of *B. orientalis* (Wolf et al., 2011). Research has shown that fern genomes undergo complex dynamic changes during evolution, with chloroplast gene transfer to mitochondria contributing to genomic diversification and influencing the adaptive capabilities of these plants under varying environmental conditions (Bock and Knoop, 2012). The findings for *B. orientalis* correspond with previous studies on ferns and lycophytes, indicating that intergenomic segment transfers are prevalent among these ancient plant lineages.

### 4.9 Phylogenetic tree analysis

The results indicate that the overall structure of the ML and BI phylogenetic trees are highly consistent, especially in the phylogenetic trees constructed based on the chloroplast genome, which align with the classification system of PPG I (Schuettpelz et al., 2016). Regardless of the method used, *B. orientalis* consistently shows the closest phylogenetic relationship with *D. crassirhizoma*, which is in line with the results from collinearity analysis, where the collinearity connections between these two species are very dense. These methods can effectively differentiate the phylogenetic relationships between *B. orientalis* and other ferns as well as lycophytes. Additionally, both methods show that the branch lengths in the mitochondrial gene-based tree are more variable than those in the chloroplast gene-based tree, which may reflect the differences in the evolutionary rates between these two organelles. In addition, we found that in the mitochondrial genome-based analysis, both methods place *S. moellendorffii* in a relatively independent branch, with its relationship to other species being not particularly close. In the ML tree, *S. moellendorffii* is positioned closer to ferns, while in the BI tree, it appears closer to lycophytes. However, the bootstrap values for both are relatively low, with confidence values of only 16 and 64, respectively. This phenomenon may be related to the absence of certain PCGs in the mitochondrial genome of *S. moellendorffii*, which makes it appear more similar to ferns. However, the cause of this positioning requires further study on *S. moellendorffii* to determine its exact phylogenetic position. This could be the result of multiple factors, such as the high variability of the mitochondrial genome, gene transfer and recombination, ancestral conservatism, and the genetic diversity within lycophytes.

The phylogenetic trees constructed using different genomic types and methods show that for the target species *B. orientalis*, both methods yield similar branching patterns and high node support, which further confirms the reliability and stability of the phylogenetic trees. Although there are some differences in the phylogenetic position of *S. moellendorffii* between the ML and BI methods, this does not mean that one method is incorrect. On the contrary, these differences highlight the advantages and disadvantages of different methods in handling data, suggesting that when constructing a phylogenetic tree, it is important to consider the applicability of the methods, the quality of the data, and the reliability of the support values. The complementary strengths and weaknesses of the two methods allow for a more accurate and in-depth understanding of the evolutionary relationships between species from different perspectives.

## 5 Conclusion

This study presents the first successful assembly and sequencing of the complete *B. orientalis* mitochondrial genome, revealing a complex structure comprising 80 contigs. It addresses a gap in the research on Blechnaceae species’ mitochondrial genome and contributes to the limited number of complete mitochondrial genome datasets available for ferns. The findings indicate that the size and GC content of the *B. orientalis* mitochondrial genome are comparable to those of previously reported fern species, with all PCGs highly conserved throughout evolution. Natural selection largely influences codon usage preference, while RNA editing events involve extensive conversions of hydrophobic amino acids. The *B. orientalis* mitochondrial genome has undergone significant rearrangements compared to closely related species and contains numerous transferred segments from the chloroplast genome. Phylogenetic trees constructed using chloroplast genome and mitochondrial genome PCGs effectively elucidate the evolutionary relationships between *B. orientalis* and other ferns and lycophytes, offering potential for molecular marker and genetic evolution studies. Furthermore, comparative analyses of mitochondrial genome characteristics, PCGs evolutionary pressures, RNA editing sites, and synteny between *B. orientalis* and ten other fern and lycophyte species provide additional insights into the differences between mitochondrial genes in ferns and lycophytes. This research provides valuable data for further investigations into Blechnaceae and enhances our understanding of the evolutionary biology of these ancient fern and lycophyte species.

## Data Availability

The complete sequence of the mitochondrial genome is accessible in the GenBank nucleotide database (https://www.ncbi.nlm.nih.gov/nucleotide/). The accession numbers is PQ143019. Additionally, the sequencing reads employed in the assembly ofthe mitochondrial genome for this study are available in the NCBI repository under the accession numbers: BioProject: PRJNA1073277, BioSample: SAMN39824912 and Sequence Read Archive (SRA) data: SRR28681840 and SRR27869072.

## Supplementary material

Supplementary Figure 1 Second-Generation Sequencing coverage depth of *B. orientalis* mitochondrial genome. The Illumina short reads were aligned to the mitochondrial genome using BWA software. The sequencing coverage was then calculated using samtools depth. Finally, a coverage plot was generated using a Python script, with the x-axis representing the mitochondrial genome length and the y-axis indicating the sequencing coverage depth.

Supplementary Figure 2 Third-Generation Sequencing coverage depth of *B. orientalis* mitochondrial genome. The Nanopore long reads from third-generation sequencing were aligned to the mitochondrial genome using BWA software. The sequencing coverage was then calculated using samtools depth. Finally, a coverage plot was generated using a Python script, with the x-axis representing the mitochondrial genome length and the y-axis indicating the sequencing coverage depth.
