# Supplementary figures and images for: The first complete mitochondrial genome assembly and comparative analysis of the fern Blechnaceae family: Blechnopsis orientalis
Source: Front Plant Sci. 2025 Mar 20;16:1534171. doi: 10.3389/fpls.2025.1534171 (PMC11965612; doi:10.3389/fpls.2025.1534171)

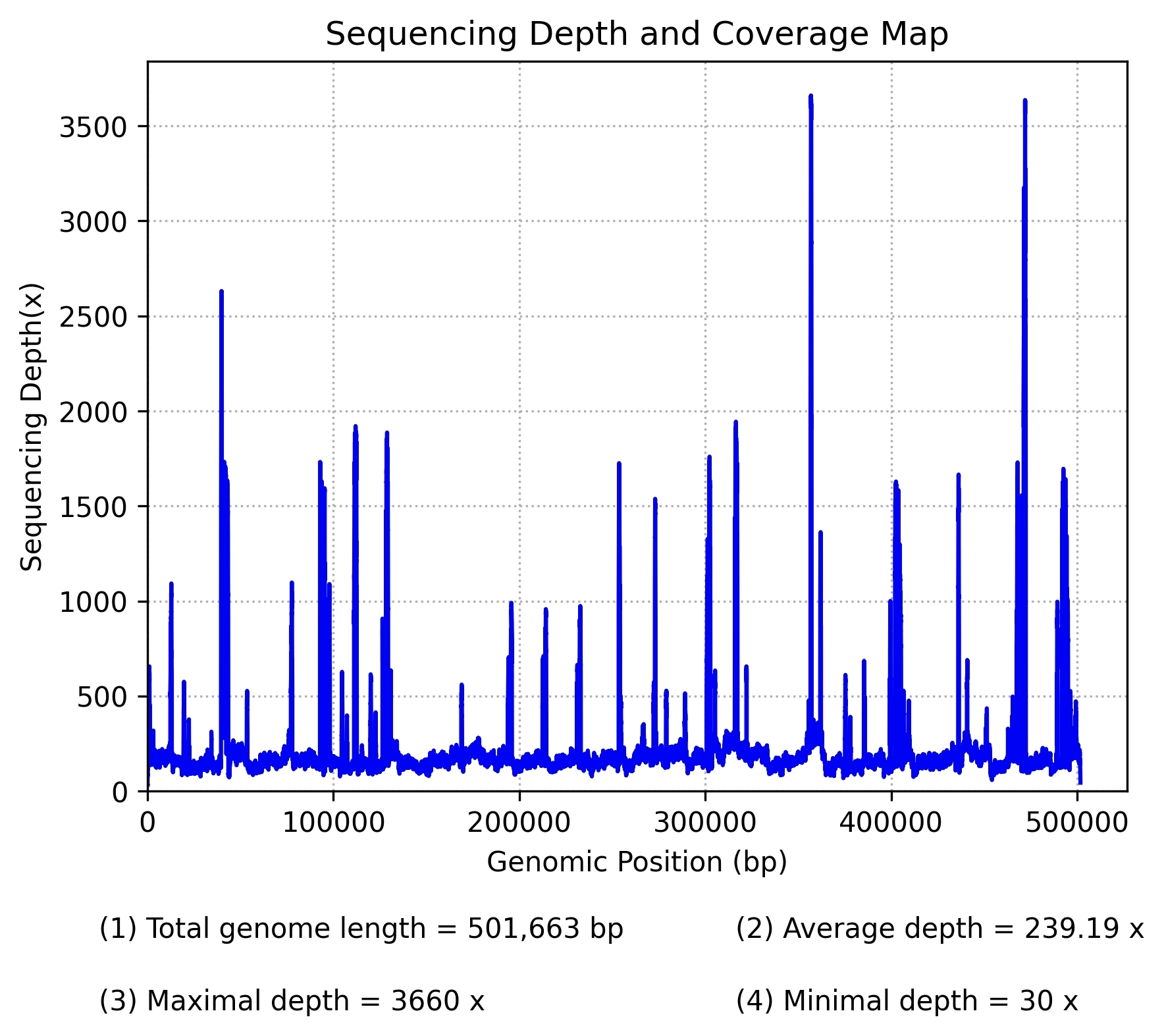

Supplement: Supplementary Figure 1 — Second-Generation Sequencing coverage depth of B. orientalis mitochondrial genome. The Illumina short reads were aligned to the mitochondrial genome using BWA software. The sequencing coverage was then calculated using samtools depth. Finally, a coverage plot was generated using a Python script, with the x-axis representing the mitochondrial genome length and the y-axis indicating the sequencing coverage depth. [file DataSheet1.zip › Supplementary Data Sheet Correction/SUPPLEMENTARY FIGURE S1.jpg]

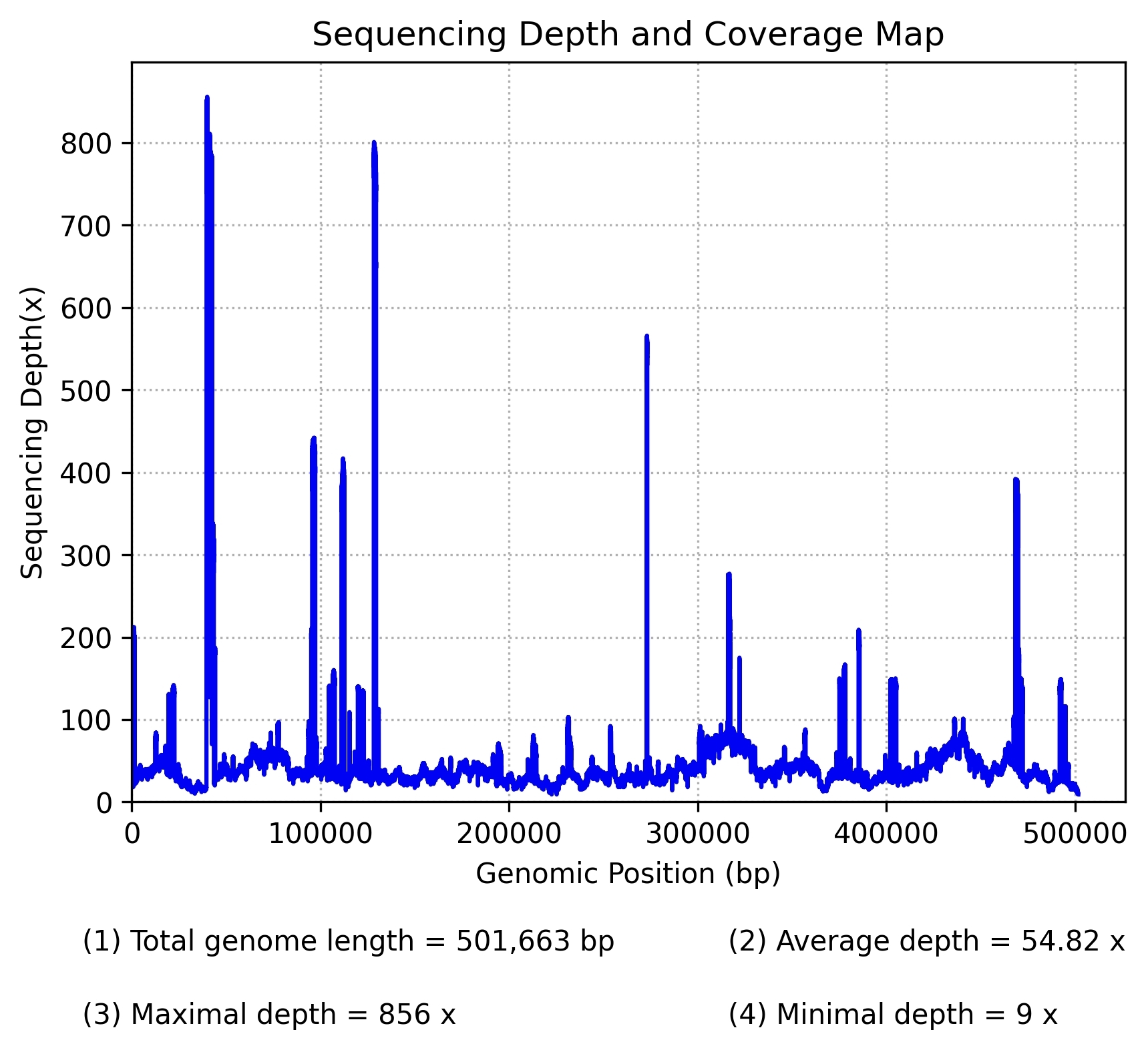

Supplement: Supplementary Figure 1 — Second-Generation Sequencing coverage depth of B. orientalis mitochondrial genome. The Illumina short reads were aligned to the mitochondrial genome using BWA software. The sequencing coverage was then calculated using samtools depth. Finally, a coverage plot was generated using a Python script, with the x-axis representing the mitochondrial genome length and the y-axis indicating the sequencing coverage depth. [file DataSheet1.zip › Supplementary Data Sheet Correction/SUPPLEMENTARY FIGURE S2.jpg]
